# On the Adaptive Design Rules of Biochemical Networks in Evolution

**Published:** 2007-02-28

**Authors:** Bor-Sen Chen, Wan-Shian Wu, Wei-Sheng Wu, Wen-Hsiung Li

**Affiliations:** 1 Lab of Control and Systems Biology, Department of Electrical Engineering, National Tsing Hua University, Hsinchu, 300, Taiwan; 2 Department of Evolution and Ecology, University of Chicago, 1101 East 57th Street, Chicago, IL 60637, U.S.A; 3 Genomics Research Center, Academia Sinica, Taipei, Taiwan

**Keywords:** evolutionary process, adaptive design rules, robust biochemical networks and S-system

## Abstract

Biochemical networks are the backbones of physiological systems of organisms. Therefore, a biochemical network should be sufficiently robust (not sensitive) to tolerate genetic mutations and environmental changes in the evolutionary process. In this study, based on the robustness and sensitivity criteria of biochemical networks, the adaptive design rules are developed for natural selection in the evolutionary process. This will provide insights into the robust adaptive mechanism of biochemical networks in the evolutionary process.

We find that if a mutated biochemical network satisfies the robustness and sensitivity criteria of natural selection, there is a high probability for the biochemical network to prevail during natural selection in the evolutionary process. Since there are various mutated biochemical networks that can satisfy these criteria but have some differences in phenotype, the biochemical networks increase their diversities in the evolutionary process. The robustness of a biochemical network enables co-option so that new phenotypes can be generated in evolution. The proposed robust adaptive design rules of natural selection gain much insight into the evolutionary mechanism and provide a systematic robust biochemical circuit design method of biochemical networks for biotechnological and therapeutic purposes in the future.

## Introduction

Robustness is a ubiquitously observed property of biological systems. It is considered to be a fundamental feature of complex evolvable systems. It is pointed out that robustness facilitates evolvability and robust traits are often selected by evolution (Kitano, 2004), i.e. complex biological systems must be robust against environmental and genetic perturbations to be evolvable. Evolution often selects traits that might enhance robustness of the organism.

The central role of biochemical networks in cellular function provides a strong motivation to search for the underlying principles of adaptive evolution of biochemical networks. In this study, in order to test whether a physiological function would prevail under a new environment or not, the robustness and sensitivity criteria are developed to measure the tolerance of the metabolite concentration values of a biochemical network in the face of environmental changes. That is, we derive necessary and sufficient conditions for the metabolite network to be preserved by natural selection in the evolutionary process.

The evolutionary analysis is based on two concepts, natural selection and evolution (Freeman and Herron, 2001). In the past, most molecular biologists and biochemists assumed that variations in biochemical networks were mainly due to historical accidents and natural selection. But the design principles of biochemical networks via natural selection in evolution are still in conceptual description, not in mathematical rules. Can these mathematical natural selection principles for biochemical networks in evolution be unraveled? The investigation of design principles of biochemical networks is in its infancy and more underlying rules remain to be discovered. In fact, robustness allows change in the structure and components of the system owing to these perturbations and disturbances, but specific functions are maintained. Hence, robustness facilitates evolvability and evolution selects robust traits (Yi et al. 2000; Kitano, 2004).

In this study, the robustness criterion based on S-system is derived as a necessary adaptive design rule of a biochemical network under natural selection. In addition, to guarantee small changes of metabolite concentration values under environmental disturbances in the evolutionary process, some sensitivity criteria are proposed as the sufficient conditions for the adaptation of a biochemical network so that it can play its proper role in the corresponding physiological system. Two adaptive design schemes of robustness improvement are developed for biochemical network evolution. One scheme is to compensate the effect of parameter variations to meet the robustness criterion easily. In this way, some redundant and self-regulatory pathways are selected by natural selection to attenuate the effect of parameter variations. The other is to enhance the system structure stability of biochemical networks to tolerate larger parameter perturbations. In this way, negative feedbacks and positive feedbacks are selected to improve structure stability. These two adaptive schemes are two design methods of biochemical networks to improve their robustness and to maintain the live function against environmental changes in the evolutionary process. The biochemical networks with improved robustness can survive under natural selection. At the same time, the sensitivities of biochemical networks to environmental disturbances are also attenuated to maintain their metabolite mechanisms for normal physiology, i.e. insusceptible to environmental disturbances for species evolution or diseases such as virus infection, with immunity for individual. They are also considered in the adaptive design rules of natural selection in evolution.

Since many solutions can meet the robustness and sensitivity criteria of natural selection, a variety of biochemical networks may survive in evolution. A variety of biochemical networks with some structural differences may arise in evolution. For example, in the TCA cycles in different species, their final products are almost the same from yeast to human, but their biochemical networks have some structural differences in intermediary biochemistry reactions. Based on the adaptive design rules of biochemical networks via natural selection, one possibility of the diversity in biochemical networks in the evolutionary process is to increase the complexity of networks through successive addition of feedback and feed-forward pathways to enhance robustness against genetic mutations and environmental perturbations (Barkai and Leibler, 1997; Alon et al. 1999; West-Eberhard, 2003).

Natural selection can select only from the mutated biochemical networks that already exist in nature and cannot instantly create a new and optimal biochemical network (or phenotype) to maintain the live function. The co-option of existing biochemical networks to new networks is one of the crucial features in evolution (Kitano, 2004). Several biochemical networks are combined through positive feedback loops and negative feedback loops so that normal cellular physiology and developmental processes can be maintained. This intrinsic robustness of a biochemical network enables co-option, so that new morphologies can be generated in the evolutionary process (Freeman, 2000; Kitano, 2004). The proposed adaptive design rule via natural selection can mimic the evolution of biochemical networks by computational simulation. Simply speaking, the evolutionary way is to improve its robustness of biochemical networks to tolerate the parameter variations and environmental variations to play their proper role in the corresponding physiological systems. The proposed robust adaptive design rules of natural selection also provide a systematic robust biochemical circuit design method of biochemical networks for drug design and robust engineered synthetic biocircuit design purposes in the future. We can use computational prediction and rational design (Altamirano et al. 2000; Johannes et al. 2005; Tsuji et al. 2006), directed evolution (May et al. 2000; Wang et al. 2000; Yuan et al. 2005) and dynamic controller (Farmer and Liao, 2000; Bulter et al. 2004) to quickly create a library of variants for artificial evolution to achieve the desired property of biochemical networks. Rational design and directed evolution are to modify the catalytic or binding property of an enzyme which corresponds to the changes of kinetic parameters *g_ij_* and *h_ij_* in the S-System model through modulating the enzyme structure and through DNA shuffling, respectively. A dynamic controller is to construct a feedback or feedforword pathway. Finally, a computational simulation example is given to illustrate the adaptive design mechanism of a biochemical network via natural selection in evolution.

## Mathematical Rules for Natural Selection

### Notations

For a vector **x** = [*x*_1_, …, *x_n_*], the *l*_2_ norm of **x** is defined as ${\left\|\text{x}\right\|}_{2}=\sqrt{x_{1}^{2}+x_{2}^{2}+\ldots +x_{n}^{2}}$. We say **x** ∈ *l*_2_, if ||**x**||_2_ < ∞. For a matrix **A** and **y** = *A***x**, the *l*_2_ – induced matrix norm is defined as ||*A*||_2_ = sup_**x**∈_*_l_*_2_ $\frac{{\left\|\text{y}\right\|}_{2}}{{\left\|\text{x}\right\|}_{2}}$, i.e. the gain from **x** to **y**. It has been shown that ||**A**||_2_ = σ*_max_* (*A*) = *max_i_* $\sqrt{\lambda_{i}(A^{T}\;A)}$ where σ*_max_* (*A*) denotes the largest singular value of *A* and *λ_i_*(*A^T^* *A*) denotes the *ith* eigenvalue of *A^T^* *A*. ||*A*||_2_ < 1 if and only if *AA^T^* < *I* i.e. *A* is contractive, where *I* is the identity matrix (Gill et al. 1991; Weinmann, 1991).

The mathematical model, robustness and sensitivity analyses of a biochemical network under natural selection in the evolutionary process are introduced at first.

## Model of a Biochemical Network

In a biochemical network, one often measures rates of reaction or influx and outflux rates of substrates, enzymes, factors or products and the rates correspond directly to changes in concentrations. The S-system model has been developed to write the reaction relationship of metabolites in differential equations in terms of their concentrations. The dynamic system of a biochemical network is described in the following S-system representation (Voit, 2000; Savageau, 2001)

(1) $$\begin{array}{l} \;\;{\dot{X}}_{1}=\alpha_{1}\prod_{j=1}^{n+m}X_{j}^{g_{1\;j}}-\beta_{1}\prod_{j=1}^{n+m}X_{j}^{h_{1\;j}}\\ \;\;\;\;\;\;\;\;\;\;\;\;\;\;\;\;\;\;\;\;\;\;\;\;\;\;\;\;\;\;\;\;\;\;\vdots \\ {\dot{X}}_{i}=\alpha_{i}\prod_{j=1}^{n+m}X_{j}^{g_{ij}}-\beta_{i}\prod_{j=1}^{n+m}X_{j}^{h_{ij}}\;\;\;\;\;\;\;\;i=2,\ldots ,\;n-1\\ \;\;\;\;\;\;\;\;\;\;\;\;\;\;\;\;\;\;\;\;\;\;\;\;\;\;\;\;\;\;\;\;\vdots \\ \;\;\;\;{\dot{X}}_{n}=\alpha_{n}\prod_{j=1}^{n+m}X_{j}^{g_{nj}}-\beta_{n}\prod_{j=1}^{n+m}X_{j}^{h_{nj}} \end{array}$$

where *X*_1_, …, *X_n_*_+_*_m_* are metabolites, such as substrates, enzymes, factors or products of a biochemical network, in which *X*_1_, …, *X_n_* denote *n* dependent variables (intermediate metabolites and products) and *X_n_*_+1_, …, *X_n_*_+_*_m_* denote the independent variables (initial reactants and enzymes). *Ẋ_i_*, the rate of change in *X_i_*, represents concentration change of a dependent variable due to production (accumulation) or degradation (clearance). Each term is the product of the rate constant, α*_i_* or β*_i_*, which is positive or zero and all dependent and independent variables that affect directly the production and degradation reaction, respectively.

Each variable *X_j_* is raised to the power of a kinetic parameter *g_ij_* and *h_ij_*, which represents that *X_j_* activates (inhibits) *X_i_* when its value is positive (negative). The rate constants α*_i_* and β*_i_* and kinetic parameters *g_ij_* and *h_ij_* are related to the characteristics of the biochemical network. The nonlinear Equation (1) describes the dynamic evolution among dependent variables. How to construct the S-system representation of a biochemical network and how to estimate its parameters from experimental data can be found in the classic textbooks (Savageau, 1976; Voit, 2000) and references therein. Recently, the nonlinear parameter estimation problem of S-systems has been efficiently solved by evolution optimization methods (Tsai and Wang, 2005; Ko et al. 2006).

Measuring directly the robustness of the nonlinear system in Equation (1) is difficult most of the time. Fortunately, the phenotype (metabolite concentration values) of a biochemical network is close to the steady state, i.e. the transient time to dynamic equilibrium is short enough in the real world and the steady state of biochemical networks can be analyzed by simple algebraic methods. Therefore, we shall focus on the robustness of a biochemical network at steady state in this paper.

Consider the steady state of biochemical network in Equation (1), i.e. the production and degradation of each dependent variable is balanced (Voit, 2000).

(2) $$\alpha_{i}\prod_{j=1}^{n+m}X_{j}^{g_{ij}}=\beta_{i}\prod_{j=1}^{n+m}X_{j}^{h_{ij}},\;\;\;\;\;\;\;\;\;\;i=1,\;2,\ldots ,\;n$$

Taking the logarithm on both sides of Equation (2), we obtain

(3) $$\begin{array}{l} \text{ln}\;\alpha_{i}+\sum_{j=1}^{n+m}g_{ij}\;\text{ln}\;X_{j}=\text{ln}\;\beta_{i}+\sum_{j=1}^{n+m}h_{ij}\;\text{ln}\;X_{j},\\ \;\;\;\;\;\;\;\;\;\;\;\;\;\;\;\;\;\;\;\;\;\;\;\;\;\;\;\;\;\;\;\;\;\;\;\;\;\;\;\;\;\;\;\;\;\;\;\;\;\;\;\;\;\;\;\;\;i=1,2,\ldots ,\;n \end{array}$$

After some rearrangements, we get

(4) $$\begin{array}{l} \sum_{j=1}^{n}(g_{ij}-h_{ij})\;\text{ln}\;X_{j}=\text{ln}\;\beta_{i}-\text{ln}\;\alpha_{i}\\ \;\;\;\;\;\;\;\;\;\;\;\;\;\;\;\;\;\;\;\;\;\;\;\;\;\;\;\;\;\;\;\;\;\;\;\;\;\;\;\;\;\;\;\;\;\;\;\;\;\;\;\;\;-\sum_{j=n+1}^{n+m}(g_{ij}-h_{ij})\;\text{ln}\;X_{j},\\ \;\;\;\;\;\;\;\;\;\;\;\;\;\;\;\;\;\;\;\;\;\;\;\;\;\;\;\;\;\;\;\;\;\;\;\;\;\;\;\;\;\;\;\;\;\;\;\;\;\;\;\;\;i=1,\;2,\ldots ,n \end{array}$$

Introduce new variables and coefficients as follows:

(5) $$y_{j}=\text{ln}\;X_{j},\;\;\;\;\;\;\;a_{ij}=g_{ij}-h_{ij},\;\;\;\;\;b_{i}-\text{ln}\left(\frac{\beta_{i}}{\alpha_{i}}\right)$$

The steady state of a biochemical system is written in *n* linear equations in terms of *n + m* variables as follows (Voit, 2000)

(6) $$\begin{array}{l} a_{11}y_{1}+a_{12}y_{2}+\cdots \cdots +a_{1n}y_{n}=b_{1}-a_{1,n+1}y_{n+1}\\ \;\;\;\;\;\;\;\;\;\;\;\;\;\;\;\;\;\;\;\;\;\;\;\;\;\;\;\;\;\;\;\;\;\;\;\;\;\;\;\;\;\;\;\;\;\;\;\;\;\;\;\;\;\;\;\;\;\;\;\;\;\;\;\;\;\;\;\;\;\;\;\;\;\;-\cdots \cdots -a_{1,n+m}y_{n+m}\\ a_{21}y_{1}+a_{22}y_{2}+\cdots \cdots +a_{2n}y_{n}=b_{2}-a_{2,n+1}y_{n+1}\\ \;\;\;\;\;\;\;\;\;\;\;\;\;\;\;\;\;\;\;\;\;\;\;\;\;\;\;\;\;\;\;\;\;\;\;\;\;\;\;\;\;\;\;\;\;\;\;\;\;\;\;\;\;\;\;\;\;\;\;\;\;\;\;\;\;\;\;\;\;\;\;\;\;\;\;\;\;\;\;\;-\cdots \cdots -a_{2,n+m}y_{n+m}\\ a_{31}y_{1}+a_{32}y_{2}\;+\cdots \cdots +a_{3n}y_{n}=b_{3}-a_{3,n+1}y_{n+1}\\ \;\;\;\;\;\;\;\;\;\;\;\;\;\;\;\;\;\;\;\;\;\;\;\;\;\;\;\;\;\;\;\;\;\;\;\;\;\;\;\;\;\;\;\;\;\;\;\;\;\;\;\;\;\;\;\;\;\;\;\;\;\;\;\;\;\;\;\;\;\;\;\;\;\;\;\;\;\;\;-\cdots \cdots -a_{3,n+m}y_{n+m}\\ \;\;\;\;\;\;\;\;\;\;\;\;\;\;\;\;\;\;\;\;\;\;\;\;\;\;\;\;\;\;\;\;\;\;\;\;\;\;\;\;\;\;\;\;\;\;\;\;\;\;\;\;\;\;\;\;\;\;\;\;\vdots \\ a_{n1}y_{1}+a_{n2}y_{2}\;+\cdots \cdots +a_{nn}y_{n}=b_{n}-a_{n,n+1}y_{n+1}\\ \;\;\;\;\;\;\;\;\;\;\;\;\;\;\;\;\;\;\;\;\;\;\;\;\;\;\;\;\;\;\;\;\;\;\;\;\;\;\;\;\;\;\;\;\;\;\;\;\;\;\;\;\;\;\;\;\;\;\;\;\;\;\;\;\;\;\;\;\;\;\;\;\;\;\;\;\;\;\;\;-\cdots \cdots -a_{n,n+m}y_{n+m} \end{array}$$

In Equation (6), the dependent variables are separated from the independent variables. Let us denote

$$\begin{array}{c} Y_{D}=\left[\begin{array}{c} y_{1}\\ \vdots \\ y_{n} \end{array}\right],\;b=\left[\begin{array}{c} b_{1}\\ \vdots \\ b_{n} \end{array}\right],\;Y_{I}=\left[\begin{array}{c} y_{n+1}\\ \vdots \\ y_{n+m} \end{array}\right]\\ A_{D}=\left[\begin{array}{ccc} a_{11} & \cdots & a_{1n}\\ \vdots & \ddots & \vdots \\ a_{n1} & \cdots & a_{nn} \end{array}\right],\;A_{I}=\left[\begin{array}{ccc} a_{1,n+1} & \cdots & a_{1,n+m}\\ \vdots & \ddots & \vdots \\ a_{n,n+1} & \cdots & a_{n,n+m} \end{array}\right] \end{array}$$

where *A_D_* denotes the system matrix of the catalytic interactions among dependent variables and *A_1_* indicates the catalytic interactions between the dependent variables *Y_D_* and the independent variables *Y_1_* (i.e. environmental medium to the metabolic system). We then obtain the steady-state equation in the nominal parameter case as follows

(7) $$A_{D}Y_{D}=b-A_{I}Y_{I}$$

## Natural Selection Criteria for Biochemical Networks in Evolution

From Equation (7), if the inverse of *A_D_* exists, then *Y_D_* can be solved uniquely. It means that the biochemical network will result in only one steady state as long as $A_{D}^{-1}$ exists. The assumption makes sense and agrees with the real biochemical networks. The steady state (or phenotype) of the biochemical network is solved as follows (Voit, 2000):

(8) $$Y_{D}=A_{D}^{-1}(b-A_{\text{I}}Y_{\text{I}})$$

Biochemical systems perform their physiological function within some local region in system parameter space in the evolutionary process. They tend to be robust to local changes in the values of the parameters that define the system in Equation (8). In the evolutionary process, suppose that some parameter variations Δα, Δβ, Δ*h*, Δ*g* and Δ*Y_I_*, which could be considered as design parameters in the evolutionary process owing to genetic mutations or environmental changes, alter the kinetic properties of a biochemical network in comparison with the nominal kinetic parameter case in Equation (7) as follows:

(9) $$\begin{array}{l} \left(A_{D}+\Delta A_{D})\;(Y_{D}+\Delta Y_{D})=(b+\Delta b\right)\\ \;\;\;\;\;\;\;\;\;\;\;\;\;\;\;\;\;\;\;\;\;\;\;\;\;\;\;\;\;\;\;\;\;\;\;\;\;\;\;\;\;\;\;\;\;\;\;\;\;\;\;\;\;\;\;\;\;\;\;\;\;\;\;\;\;\;-(A_{I}+\Delta A_{I})\;(Y_{I}+\Delta Y_{I}) \end{array}$$

where the parameter variations of the biochemical network are defined by

$$\begin{array}{l} \Delta A_{D}=\left[\begin{array}{ccc} \Delta a_{11} & \cdots & \Delta a_{1n}\\ \vdots & \Delta a_{ij} & \vdots \\ \Delta a_{n1} & \cdots & \Delta a_{nn} \end{array}\right]\\ \;\;\;\;\;\;\;\;\;\;\;\;\;\;\;\;\;\;\;\;\;=\left[\begin{array}{ccc} \Delta g_{11}\;-\;\Delta h_{11} & \cdots & \Delta g_{1n}\;-\;\Delta h_{1n}\\ \vdots & \Delta g_{ij}\;-\;\Delta h_{ij} & \vdots \\ \Delta g_{n1}\;-\;\Delta h_{n1} & \cdots & \Delta g_{nn}\;-\;\Delta h_{nn} \end{array}\right],\\ \Delta Y_{D}\;=\;\left[\begin{array}{c} \Delta y_{1}\\ \vdots \\ \Delta y_{n} \end{array}\right]\\ \Delta A_{I}\;=\;[\begin{array}{cc} \Delta g_{1,n\;+\;1}\;-\;\Delta h_{1,n\;+\;1} & \cdots \\ \vdots & \Delta g_{i,n\;+\;j}\;-\;\Delta h_{i,n\;+\;j}\\ \Delta g_{n,n\;+\;1}\;-\;\Delta h_{n,n\;+\;1} & \cdots \end{array}\\ \;\;\;\;\;\;\;\;\;\;\;\;\;\;\;\;\;\;\begin{array}{c} \Delta g_{1,n\;+\;m}\;-\;\Delta h_{1,n\;+\;m}\\ \vdots \\ \Delta g_{n,n\;+\;m}\;-\;\Delta h_{n,n\;+\;m} \end{array}],\;\Delta Y_{I}\;=\;\left[\begin{array}{c} \Delta y_{n\;+\;1}\\ \vdots \\ \Delta y_{n\;+\;m} \end{array}\right],\\ \Delta b\;=\;\left[\begin{array}{c} \Delta b_{1}\\ \vdots \\ \Delta b_{n} \end{array}\right] \end{array}$$

Δ*A_D_* denotes the kinetic parameter variations owing to the kinetic parameter variations Δ*g_ij_* and Δ*h_ij_* within dependent variables; Δ*b* denotes the parameter variations owing to rate constant variations and Δ*A_I_* denotes the kinetic parameter variations owing to the kinetic parameter variations Δ*g_ij_* and Δ*h_ij_* between independent and dependent variables. Δ*Y_I_* denotes the concentration variations of environmental factors. Δ*Y_D_* is a perturbation effect which may lead to new steady state. *Y_D_*+Δ*Y_D_* may own a little difference from the nominal phenotype *Y_D_* if these variations could be tolerated by the biochemical network.

The robustness analysis of a biochemical network in this study is to check the tolerance for kinetic parameter variations with respect to the maintenance of normal physiological function of perturbed biochemical networks in the evolutionary process. First, the Δ*A_D_* will influence the existence of the steady state. Then, the variations Δ*b*, Δ*A_I_* and Δ*Y_I_* will influence the sensitivity of biochemical networks to the environmental variations in the evolutionary process. The effects of these parameter variations (i.e. the design parameter space in evolution) on the biochemical network at the steady state (i.e. *Ẋ_i_* = 0) will be discussed in the following paragraphs.

Equation (9) is equivalent to

(10) $$\begin{array}{l} A_{D}\;(I+A_{D}^{-1}\Delta A_{D})\;(Y_{D}+\Delta Y_{D})=(b+\Delta b)\\ \;\;\;\;\;\;\;\;\;\;\;\;\;\;\;\;\;\;\;\;\;\;\;\;\;\;\;\;\;\;\;\;\;\;\;\;\;\;\;\;\;\;\;\;\;\;\;\;\;\;\;\;\;\;\;\;\;\;\;\;\;\;\;\;\;\;\;\;\;\;\;\;\;\;\;\;\;\;\;-(A_{I}+\Delta A_{I})\;(Y_{I}+\Delta Y_{I}) \end{array}$$

By the similar analysis from Equation (7) to Equation (8), one can show that the condition that the system in Equation (10) can be solved uniquely is the existence of the inverse of $\left(I+A_{D}^{-1}\Delta A_{D}\right)$. It has been shown that if the following robustness criterion holds (Gill et al. 1991; Weinmann, 1991; Nobel and Daniel, 1998; Chen et al. 2005)

(11) $$\|\;A_{D}^{-1}\;\Delta A_{D}\;{\|}_{2}<1\;\;\;\;\;\;\;or\;\;\;\;\;\;\;\Delta A_{D}\Delta A_{D}^{T}<A_{D}A_{D}^{T}$$

the inverse ${\left(I+A_{D}^{-1}\Delta A_{D}\right)}^{-1}$ exists and the phenotype (steady state) of the perturbed biochemical network in Equation (10) is uniquely solved as follows

(12) $$\begin{array}{l} Y_{D}+\Delta Y_{D}={\left(I+A_{D}^{-1}\Delta A_{D}\right)}^{-1}\;A_{D}^{-1}[(b+\Delta b)\\ \;\;\;\;\;\;\;\;\;\;\;\;\;\;\;\;\;\;\;\;\;\;\;\;\;\;\;\;\;\;\;-(A_{I}+\Delta A_{I})\;(Y_{I}+\Delta Y_{I})] \end{array}$$

The physical meaning of Equation (11) and (12) is that if the inverse ${\left(I+A_{D}^{-1}\Delta A_{D}\right)}^{-1}$ exists, the phenotype can be preserved with some variation under this parameter variation Δ*A_D_*, i.e. if the robustness criterion in Equation (11) is satisfied, the parameter variations Δ*A_D_* can be tolerated by the system structure of the biochemical network *A_D_* in the evolutionary process and the biochemical network tends to be robust to local changes in the values of parameters that define the system. Otherwise, the parameters reach a threshold beyond which the behavior of the biochemical network changes dramatically and the phenotype may cease to exist, i.e. the individuals with parameter variations (design parameters in evolution) that violate the robustness criterion in Equation (11) will be eliminated by natural selection. Therefore, the perturbed biochemical network should satisfy the robustness criterion in order to guarantee the existence of its dynamic equilibrium (for the normal physiological function) in the evolutionary process. Because the violation of Equation (11) means a lethal perturbation, it is the necessary condition to survive under natural selection. From the robustness criterion in Equation (11), natural selection favors the perturbed biochemical networks with small perturbations Δ*A_D_* $\Delta A_{D}^{T}$ or a large system structure stability matrix *A_D_* $A_{D}^{T}$ so that the robustness criterion is not violated. A biochemical network with redundancy and self-regulation can attenuate perturbations Δ*A_D_* and a biochemical network with adequate negative feedbacks can increase *A_D_* $A_{D}^{T}$ to tolerate large parameter variations in the evolutionary process. These robust adaptive designs are favored by natural selection in the evolutionary process of biochemical networks. This is why there are so much redundancy due to duplicated genes, modularity, self-regulation and feedback circuits in the biochemical networks in nature (Isaacs et al. 2003; Langkjaer et al. 2003; Kellis et al. 2004; Teichman and Babu, 2004).

However, the satisfaction of the robustness criterion in Equation (11), i.e. the parameter variations Δ*A_D_* ${\mathrm{\Delta A}}_{D}^{T}$ is bounded by the system structure matrix *A_D_* $A_{D}^{T}$ does not always mean the perturbed biochemical network will survive in evolution because it only guarantees the existence of the steady state. But the phenotype (steady state) may be far from the nominal value for the normal physiological function. In order to play its proper role in the corresponding physiological system, its metabolite concentration values should not change too much from the nominal value. In this situation, the biochemical network should be less sensitive to the other parameter variations and environmental changes. This is the sufficient condition for natural selection for a perturbed biochemical network to survive under natural selection. In the above robust analysis, we only discussed the effect of kinetic parameter variations Δ*A_D_* on the existence of the steady state of a biochemical network. Now, let us consider the sensitivities to the variations of the other parameters Δ*b*, Δ*A_I_* and the change of the environment Δ*Y_I_* in the evolutionary process.

The changes Δ*b*, Δ*Y_I_*, and Δ*A_I_* will influence the variations of steady states *Y_D_*. Their effects on *Y_D_* have been discussed by the sensitivity analysis of biochemical network (Savageau, 1971; [Bibr b22-ebo-03-27][Bibr b23-ebo-03-27]; Voit, 2000), i.e.

(13) $$\frac{\Delta Y_{D}}{\Delta b}=A_{D}^{-1},\;\;\;\frac{\Delta Y_{D}}{\Delta Y_{I}}=-A_{D}^{-1}\;A_{I},\;\;\;\frac{\Delta Y_{D}}{\Delta A_{I}}=-A_{D}^{-1}\;Y_{I}$$

In order to tolerate the variations Δ*b*, Δ*Y_I_* and Δ*A_I_* to preserve the phenotype of the biochemical network in the evolutionary process, the sensitivities in Equation (13) should be below some values as follows

(14) $${\left\|\frac{\Delta Y_{D}}{\Delta b}\right\|}_{2}\leq s_{1},\;\;\;{\left\|\frac{\Delta Y_{D}}{\Delta Y_{I}}\right\|}_{2}\leq s_{2},\;\;\;{\left\|\frac{\Delta Y_{D}}{\Delta A_{I}}\right\|}_{2}\leq s_{3}$$

where *s*_1_, *s*_2_ and *s*_3_ are some small sensitivity values so that the phenotypes of perturbed biochemical networks would not change too much in comparison with the nominal values in Equation (13) and can be favored by natural selection, i.e. the sensitivity criterion in Equation (14) can be considered as the sufficient condition of natural selection for biochemical network evolution. In general, the sensitivities *s*_1_, *s*_2_ and *s*_3_ are chosen as the sensitivities at the nominal case, because the nominal (healthy) biochemical network is less-sensitive to parameter variations and environmental changes. Based on Equation (13), Equation (14) can be written in the following equivalent form,

(15) $$\begin{array}{l} I\leq s_{1}^{2}\;A_{D}\;A_{D}^{T},\;\;\;\;\;\;A_{I}\;A_{I}^{T}\leq s_{2}^{2}\;A_{D}\;A_{D}^{T},\\ \;\;\;\;\;\;\;\;\;\;\;\;\;\;\;\;\;\;Y_{I}\;Y_{I}^{T}\leq s_{3}^{2}\;A_{D}\;A_{D}^{T} \end{array}$$

That is, Equation (15) determines the ranges of the sensitivities of *Y_D_* to parameter variations and environmental changes by natural selection in the evolutionary process. For a functional biochemical network, it should satisfy the sensitivity criteria in Equation (15) to confine the metabolite concentration values not to be changed too much. Hence, the steady state (phenotype) of a biochemical network can be preserved while exposing the parameter variations and environmental changes to natural selection in the evolutionary process. This can be considered as a sufficient condition for survival for the biochemical network.

The robustness criterion in Equation (11) and the sensitivity criterion in Equation (15) are together considered as the criteria of natural selection in evolution. If one of them is violated, it will lead to the dysfunction of the biochemical network and the perturbed biochemical network will be eliminated by natural selection. Therefore, the robustness criterion in Equation (11) and the sensitivity criterion in Equation (15) could be considered as the adaptive design rules of biochemical networks by natural selection in the evolutionary process. The specifications of sensitivities *s_i_*, *i* = 1, 2, 3 in Equation (15) are species by species. In general, these sensitivities should be small in order to avoid too much influence from the environmental disturbances in the evolutionary process.

### Remark 1

- The equality for robustness criterion in Equation (11) can not hold because it may make $I+A_{D}^{-1}\;\Delta A_{D}$ singular (for example $A_{D}^{-1}$ Δ*A_D_*=−*I*) and the steady will cease to exist. However, the equality could hold in Equation (14) because we do not want the sensitivities of perturbed systems to be larger than the sensitivities of the nominal system, which has no singular problem.
- Actually, the sensitivity matrices in Equation (13) hold if all the perturbations Δ*b*, Δ*Y_I_*, Δ*A_I_* are very small (Voit, 2000) (it was originally derived by $\frac{\partial Y_{D}}{\partial b}=A_{D}^{-1}$, $\frac{\partial Y_{D}}{\partial Y_{I}}=-A_{D}^{-1}\;A_{I}$, $\frac{\partial Y_{D}}{\partial A_{I}}=-A_{D}^{-1}\;Y_{I}$. For the convenience of discussion on perturbations, it was modified to the form in Equation (13)). If some perturbations are large, the equalities may be violated. One proposition of Theory of Evolution is that “Gradual evolution results from small genetic changes that are acted upon by natural selection” (Freeman and Herron, 2001). Obviously, in evolutionary process, Δ*b*, Δ*Y_I_*, Δ*A_I_* are all assumed to be small in every change. In this situation, the equalities in Equation (13) always hold.
- The assumption that the three sensitivity inequalities in Equation (14) all hold for natural selection is based on the fact that biochemical networks are the backbones of physiological systems and can not be too sensitive to environmental changes especially for some core (conserved) biochemical networks. If some sensitivity criteria in Equation (14) are relaxed, i.e. some of inequalities in Equation (14) are violated, the phenotypes with changes to some environmental variation will also be favored by natural selection. In this situation, the phenotypes of biochemical networks are much influenced by environmental variation that they may be more adaptive to the environmental changes in the evolutionary process. In this case, new phenotypes are more easily generated to adapt the new environment. They will be discussed in the sequel.

## Computational Examples

An example is given below to illustrate the mathematical adaptive design rules of biochemical networks by natural selection in the evolutionary process. Consider the following biochemical network (Savageau, 1976; Voit, 2000).

(16) $$\begin{array}{ll} {\dot{X}}_{1}=10X_{2}^{-0.1}\;X_{3}^{-0.05}\;X_{4}-5X_{1}^{0.5}, & X_{1}(0)=0.2\\ {\dot{X}}_{2}=2X_{1}^{0.5}-1.44X_{2}^{0.5}, & X_{2}(0)=0.5\\ {\dot{X}}_{3}=3X_{2}^{0.5}-7.2X_{3}^{0.5}, & X_{3}(0)=0.1,\\ & X_{4}=0.75 \end{array}$$

**Figure 1.**
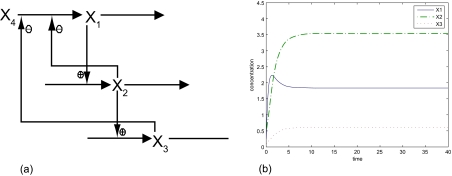
The biochemical network in Equation (16) and its time responses.

The biochemical network and its time responses are shown in Figure 1.

Suppose the biochemical network suffers the following four parameter variations due to genetic mutations in the evolutionary process.

(17) $$\begin{array}{l} \Delta A_{D_{1}}=\left[\begin{array}{ccc} 0 & 0 & 0\\ 0 & 0.2826 & 0.3\\ 0 & 0 & 0.0914 \end{array}\right],\\ \Delta A_{D_{2}}=\left[\begin{array}{ccc} 0.3 & 0 & 0.05\\ 0 & 0 & 0\\ 0 & 0 & 0 \end{array}\right] \end{array}$$

(18) $$\begin{array}{l} \Delta A_{D_{3}}=[\begin{array}{ccc} 0.04675 & 0 & 0\\ 0 & -0.11 & -0.1\\ -0.31 & 0.06494 & 0.0914 \end{array}],\\ \Delta A_{D_{4}}=\left[\begin{array}{ccc} 0 & 0 & 0\\ 0 & 0 & -0.43\\ 0 & 0 & 0 \end{array}\right] \end{array}$$

The biochemical network in Equation (16) is then perturbed to the following four networks:

(19) $$(I)\{\begin{array}{ll} {\dot{X}}_{1}=10X_{2}^{-0.1}\;X_{3}^{-0.05}\;X_{4}-5X_{1}^{0.5}, & X_{1}(0)=0.2\\ {\dot{X}}_{2}=2X_{1}^{0.5}\;\underline{X_{3}^{0.3}}-1.44X_{2}^{\underline{0.2174}}, & X_{2}(0)=0.5\\ {\dot{X}}_{3}=3X_{2}^{0.5}-7.2X_{3}^{\underline{0.4086}}, & X_{3}(0)=0.1,\\ & X_{4}=0.75 \end{array}$$

(20) $$(II)\{\begin{array}{ll} {\dot{X}}_{1}=10X_{2}^{-0.1}\;X_{3}^{\underline{0}}X_{4}-5X_{1}^{\underline{0.2}}, & X_{1}(0)=0.2\\ {\dot{X}}_{2}=2X_{1}^{0.5}-1.44X_{2}^{0.5}, & X_{2}(0)=0.5\\ {\dot{X}}_{3}=3X_{2}^{0.5}-7.2X_{3}^{0.5}, & X_{3}(0)=0.1,\\ & X_{4}=0.75 \end{array}$$

(21) $$(III)\{\begin{array}{l} {\dot{X}}_{1}=10X_{2}^{-0.1}\;X_{3}^{-0.05}\;X_{4}-5X_{1}^{\underline{0.45325}},\\ \;\;\;\;\;\;\;\;\;\;\;\;\;\;\;\;\;\;\;\;\;\;\;\;\;\;\;\;\;\;\;\;\;\;\;\;\;\;\;\;\;\;\;\;\;\;\;\;\;\;\;\;\;\;\;\;\;\;\;\;\;\;\;\;X_{1}(0)=0.2\\ {\dot{X}}_{2}=2X_{1}^{0.5}\;\underline{X_{3}^{-0.1}}-1.44X_{2}^{\underline{0.61}},\\ \;\;\;\;\;\;\;\;\;\;\;\;\;\;\;\;\;\;\;\;\;\;\;\;\;\;\;\;\;\;\;\;\;\;\;\;\;\;\;\;\;\;\;\;\;\;\;\;\;\;\;\;X_{2}(0)=0.5\\ {\dot{X}}_{3}=3X_{2}^{\underline{0.56494}}\;\underline{X_{1}^{-0.31}}-7.2X_{3}^{\underline{0.4086}},\\ \;\;\;\;\;\;\;\;\;\;\;\;\;\;\;\;\;\;\;\;\;\;\;\;X_{3}(0)=0.1,\;X_{4}=0.75 \end{array}$$

(22) $$(IV)\{\begin{array}{ll} {\dot{X}}_{1}=10X_{2}^{-0.1}\;X_{3}^{-0.05}\;X_{4}-5X_{1}^{0.5}, & X_{1}(0)=0.2\\ {\dot{X}}_{2}=2X_{1}^{0.5}\;\underline{X_{3}^{-0.43}}-1.44X_{2}^{0.5}, & X_{2}(0)=0.5\\ {\dot{X}}_{3}=3X_{2}^{0.5}-7.2X_{3}^{0.5}, & X_{3}(0)=0.1,\\ & \;\;\;\;\;\;X_{4}=0.75 \end{array}$$

**Figure 2.**
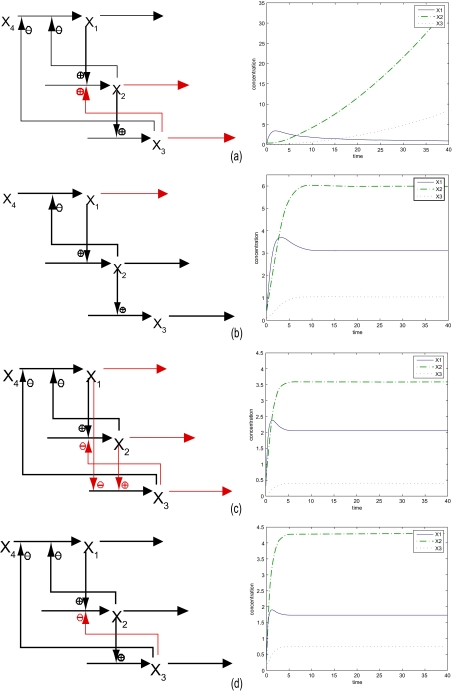
The perturbed biochemical networks and their time responses. (**a**) Biochemical network (I) in Equation (19) and its time responses. (**b**) Biochemical network (II) in Equation (20) and its time responses. (**c**) Biochemical network (III) in Equation (21) and its time responses. (**d**) Biochemical network (IV) in Equation (22) and its time responses. Biochemical network (I) is lethal, biochemical network (II) is not lethal but sensitive to environmental disturbances and may be eliminated by natural selection, and biochemical networks (III) and (IV) are robust to intrinsic and extrinsic variations and are favored by natural selection.

The perturbed biochemical networks and their time responses are shown in Figure 2. Suppose the sensitivity criteria *s*_1_, *s*_2_ and *s*_3_ in Equation (15) are chosen as the sensitivities of the nominal biochemical network in Equation (13), i.e. $s_{1}={\left\|A_{D}^{-1}\right\|}_{2}$, $s_{2}={\left\|A_{D}^{-1}A_{I}\right\|}_{2}$ and ${\text{s}}_{3}={\left\|A_{D}^{-1}Y_{I}\right\|}_{2}$. That is to say, the perturbed networks to be selected by natural selection should have less sensitivities than the nominal biochemical network. By the adaptive design rules based on robustness and sensitivity criteria, the perturbed biochemical network (I) in Equation (19) violates the robustness criterion in Equation (11) and the parameter variations Δ*A*_*D*_1__ can not be tolerated by the biochemical network. In this situation, the biochemical network (I) will be eliminated by natural selection without consideration of sensitivities. More precisely, the set of parameter variations Δ*A*_*D*_1__ due to mutations is lethal. Though the biochemical network (II) in Equation (20) satisfies the robustness criterion in Equation (11), its steady state is farther from nominal value and violates sensitivity criterion in Equation (15). It means that the phenotype of biochemical network (II) is easier (more sensitive) to be destroyed while exposing to environmental disturbances, i.e. Δ*A_I_*, Δ*b* or Δ*Y_I_* due to environmental changes. Hence, there is a large probability that the biochemical network (II) will be eliminated by natural selection. More precisely, the parameter variations due to mutations Δ*A_D_*__2__ are not lethal, but the biochemical network is susceptible to environmental disturbances for species evolution or diseases such as virus infection, i.e. less immunity for individuals.

From Figure 2, it is seen that the steady states, *Y_D_*__3__ + Δ*Y_D_*__3__ and *Y*_*D*_4__ + Δ*Y_D_*__4__, of biochemical network (III) in Equation (21) and biochemical network (IV) in Equation (22), respectively, are all close to the nominal values of the steady state in the nominal biochemical network of Equation (16) in Figure 1. In addition, the robustness criterion in Equation (11) and the sensitivity criterion in Equation (15) are all satisfied so that the variations Δ*A*_*D*_3__ and Δ*A*_*D*_4__, Δ*b*_3_ and Δ*b*_4_, Δ*A*_*I*_3__ and Δ*A*_*I*_4__ as well as environmental disturbances Δ*Y*_*I*_3__ and Δ*Y*_*I*_4__ do not affect the normal function of the biochemical networks too much. In other words, the biochemical networks (III) and (IV) are robust to intrinsic parameter variations and less sensitive to environmental variations, so that the two biochemical networks must be more favored by natural selection. In the next generation, the other perturbed biochemical networks will be selected by natural selection with the same procedure. This co-option of existing biochemical networks to new networks by natural selection is considered one of the crucial features in the evolutionary procedure. Several biochemical networks combined with negative and positive feedback loops are robust against parameter variations and environmental disturbances so that normal cellular physiological and developmental processes can be maintained. This intrinsic robustness and sensitivity of the biochemical network enable co-option, so that the new phenotypes can be generated by natural selection (Kitano, 2004). Therefore, the robustness criterion in Equation (11) and sensitivity criterion in Equation (15) can be viewed as the mathematical adaptive design rules of natural selection.

From Table 1, we can find that the sensitivities of biochemical network (III) and biochemical network (IV) are both smaller than the nominal one. That is, there is a high probability that the mutated biochemical networks with smaller sensitivities *s*_1_, *s*_2_ and *s*_3_ can prevail under natural selection. The sensitivities of biochemical networks in Equations (16), (19), (20), (21) and (22) are listed in Table 1.

## Diversity of Biochemical Network within Organisms or Individuals in Evolution

There are many perturbed biochemical networks that can satisfy the adaptive design rules of natural selection in Equations (11) and (15) to survive in the evolutionary process. If they are all selected by natural selection, there will be some differences in phenotype (see Equation (12)) among these selected biochemical networks. However, as the values of parameters continue to change, they reach a threshold (i.e. the robustness criterion in Equation (11) is violated) beyond which the behavior of the biochemical network changes dramatically. It may thus settle in a new local region of another steady state with a different set of behaviors, or it may become completely dysfunctional and not survive under natural selection in evolution.

After several generations in the evolutionary process, due to co-option of existing biochemical networks to new networks, diversities of biochemical networks with conserved physiological function but with different structures will be developed (Freeman, 2000). However, if the requirements on the robustness in Equations (11) and the sensitivity in Equations (15) are more strict (or more conservative), only few solutions (or structures) can be selected by the natural selection to meet these requirements. This is the reason why a conserved core biochemical network has less diversity (Kitano, 2004). For examples, in the evolutionary process of the TCA cycle, the pentosephosphate pathway and the glycolysis pathway in different species, their final products are almost the same from yeast to human. However, their biochemical networks have some differences in intermediary biochemistry reactions. From the simulation examples of perturbed biochemical networks in Equation (19), (20), (21) and (22), the perturbed biochemical networks (III) and (IV) in Equations (21) and (22), which are shown in Figure 2c and 2d, respectively, can be seen as the diversities of the biochemical network in the evolutionary process.

### Remark 2

**Figure 3.**
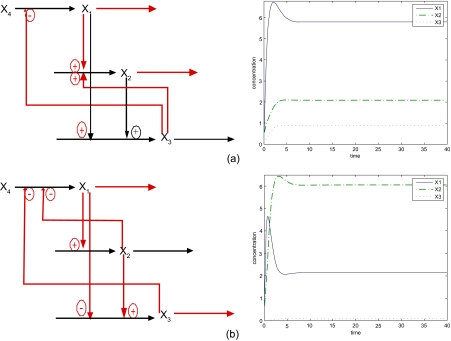
The perturbed biochemical networks and their time responses. (**a**) Biochemical network (V) in Equation (24) and its time responses. (**b**) Biochemical network (VI) in Equation (25) and its time responses. The biochemical network V will adapt to the environment with large variation Δ*Y_I_* in evolution. The biochemical network will adapt to an environment with large variation in Δ*b* and Δ*A_I_*.

Since the violation of sensitivity criteria of natural selection in Equation (15) is not lethal, the relaxation of some sensitivity inequalities in natural selection will make biochemical networks more easily adapt to new environmental changes. For example in Figure 3a and 3b, suppose the biochemical network suffers Δ*A_D_*__5__ and Δ*A_D_*__6__, respectively, due to genetic mutations in the evolutionary process in Equation (23), the biochemical network in Equation (16) is then perturbed to the (V) and (VI) network in Equation(24) and (25), respectively. In the former case, the second sensitivity criterion in Equation (15) is violated. The biochemical network will adapt to the environment with large variation Δ*Y_I_* in evolution. In the latter case, the first and third sensitivity criteria are relaxed. The biochemical network will adapt to an environment with large variation in Δ*b* and Δ*A_I_*. The sensitivities of biochemical networks in Equation (24) and (25) are listed in Table 2.

(23) $$\begin{array}{l} \Delta A_{D5}=\left[\begin{array}{ccc} 0.25 & 0.1 & -0.2\\ -0.25 & -0.5 & 0.25\\ 0.25 & 0 & 0 \end{array}\right],\\ \Delta A_{D6}=\left[\begin{array}{ccc} -0.25 & -0.15 & -0.2\\ 0.25 & 0 & 0\\ -0.25 & -0.25 & 0.25 \end{array}\right] \end{array}$$

(24) $$(V)\{\begin{array}{ll} {\dot{X}}_{1}=10X_{2}^{\underline{0}}\;X_{3}^{\underline{-0.25}}\;X_{4}-5X_{1}^{\underline{0.25}}, & X_{1}(0)=0.2\\ {\dot{X}}_{2}=2X_{1}^{\underline{0.25}}\;\underline{X_{3}^{0.25}}-1.44X_{2}^{\underline{1}}, & X_{2}(0)=0.5\\ {\dot{X}}_{3}=3X_{2}^{0.5}\;\underline{X_{1}^{0.25}}-7.2X_{3}^{0.5}, & X_{3}(0)=0.1,\\ & X_{4}=0.75 \end{array}$$

(25) $$(VI)\{\begin{array}{ll} {\dot{X}}_{1}=10X_{2}^{\underline{-0.25}}\;X_{3}^{\underline{-0.25}}\;X_{4}-5X_{1}^{\underline{0.75}}, & X_{1}(0)=0.2\\ {\dot{X}}_{2}=2X_{1}^{\underline{0.75}}-1.44X_{2}^{0.5}, & X_{2}(0)=0.5\\ {\dot{X}}_{3}=3X_{2}^{\underline{0.25}}\;\underline{X_{1}^{-0.25}}-7.2X_{3}^{\underline{0.25}}, & X_{3}(0)=0.1,\\ & X_{4}=0.75 \end{array}$$

## Conclusion

From the biochemical network evolution point of view, a biochemical network should be robust enough to maintain its proper role in the physiological system under parameter variations due to mutations and environmental disturbances. On the basis of stability robustness and less sensitivity to the effects of genetic mutations and environmental variations, the proposed design rules (robustness and sensitivity criteria) are developed as the underlying mathematical adaptive design principles of natural selection in the biochemical network evolution. That is, in the evolutionary process, organism enhances the structure stability by feedback or feedforward circuits to improve the robustness of a biochemical network to tolerate parameter variations or compensates the effect of external or internal perturbations. The self-regulation and redundancy are of this kind of robust design favored by natural selection in the evolutionary process (Becskei and Serrano, 2000; Issacs et al. 2003). Therefore, in the evolutionary process of biochemical networks, robustness is the maintenance of specific functionalities of the network against perturbations, and it often requires the biochemical network to change its mode of operation in a flexible way. In other words, robustness allows changes in the structure and components of the biochemical network (the so-called adaptive design) owing to parameter perturbation and environmental disturbances, but specific functions are maintained. Because there are several solutions that can meet the robustness and sensitivity criteria of the adaptive design rules by natural selection, this is the origin of diversities of the biochemical networks within organisms or individuals in evolution. These adaptive design rules incurring robustness of biochemical networks actually facilitate evolution, and evolution favors robust biochemical network. Therefore, requirements for robustness and evolvability are similar in biochemical networks. This implies that there are architectural requirements for biochemical networks to be evolvable, which essentially require biochemical network to be robust against genetic perturbation and environmental disturbance (Kitano, 2004).

In this study, a mathematical modeling is provided for the robust adaptive design mechanism of biochemical networks in evolution. As the parameter variations of biochemical networks continue to increase in some local region, they will reach a threshold (i.e. the robustness criterion of natural selection is violated) beyond which the behavior of biochemical network will change dramatically. It may then settle in a local region of another steady state (phenotype) with a different set of behaviors, or it may become dysfunctional and can not persist in evolution. Furthermore, if some sensitivity criteria of natural selection are relaxed, biochemical networks will be more susceptible to the corresponding environmental changes and turn out to be preferred in a new environment.

By using in silico examples, an adaptive design rule of a biochemical network is revealed by the S-system dynamic model to illustrate the natural selection and diversification in evolution from the robustness and sensitivity point of views. This provides much insight into the evolutionary mechanism of biochemical networks from the system perspective, and the proposed deign rules by natural selection will highlight the robust biochemical circuit design methods of biochemical networks via inserting the binding sites of transcription factors to the regulated genes (Tsai et al. 2006) for biotechnological and therapeutic purpose in future (Savageau, 2001; Hasty et al. 2002; Kitano, 2002ab; Hood et al. 2004).

## Figures and Tables

**Table 1:** The sensitivities of nominal and perturbed biochemical networks.

**Biochemical networks**	***s*_1_**	***s*_2_**	***s*_3_**
Biochemical network in Equation (16)	3.4191	2.6647	2.5643
Biochemical network (I) in Equation (19)	217.05	140.14	162.78
Biochemical network (II) in Equation (20)	6.0274	5.7735	4.5206
Biochemical network (III) in Equation (21)	2.8203	2.3801	2.1152
Biochemical network (IV) in Equation (22)	2.276	2.1635	1.707

**Table 2:** The sensitivities of nominal and perturbed biochemical networks.

**Biochemical networks**	***s*_1_**	***s*_2_**	***s*_3_**
Biochemical network in Equation (16)	3.4191	2.6647	2.5643
Biochemical network in Figure 3a	3.0248	3	2.2686
Biochemical network in Figure 3b	4.1534	1.4967	3.1151
